# Eicosapentaenoic Acid and Docosahexaenoic Acid in Whole Blood Are Differentially and Sex-Specifically Associated with Cardiometabolic Risk Markers in 8–11-Year-Old Danish Children

**DOI:** 10.1371/journal.pone.0109368

**Published:** 2014-10-15

**Authors:** Camilla T. Damsgaard, Maj B. Eidner, Ken D. Stark, Mads F. Hjorth, Anders Sjödin, Malene R. Andersen, Rikke Andersen, Inge Tetens, Arne Astrup, Kim F. Michaelsen, Lotte Lauritzen

**Affiliations:** 1 Department of Nutrition, Exercise and Sports, Faculty of Science, University of Copenhagen, Frederiksberg, Denmark; 2 Department of Kinesiology, University of Waterloo, Waterloo, Alberta, Canada; 3 Department of Clinical Biochemistry, Copenhagen University Hospital, Gentofte, Denmark; 4 Division of Nutrition, National Food Institute, Technical University of Denmark, Søborg, Denmark; Inserm, France

## Abstract

n-3 long-chain polyunsaturated fatty acids improve cardiovascular risk markers in adults. These effects may differ between eicosapentaenoic acid (EPA, 20∶5n-3) and docosahexaenoic acid (DHA, 22∶6n-3), but we lack evidence in children. Using baseline data from the OPUS School Meal Study we 1) investigated associations between EPA and DHA in whole blood and early cardiometabolic risk markers in 713 children aged 8–11 years and 2) explored potential mediation through waist circumference and physical activity and potential dietary confounding. We collected data on parental education, pubertal stage, 7-day dietary records, physical activity by accelerometry and measured anthropometry, blood pressure, and heart rate. Blood samples were analyzed for whole blood fatty acid composition, cholesterols, triacylglycerol, insulin resistance by the homeostatic model of assessment (HOMA-IR), and inflammatory markers. Whole blood EPA was associated with a 2.7 mmHg (95% CI 0.4; 5.1) higher diastolic blood pressure per weight% EPA, but only in boys. Heart rate was negatively associated with both EPA and DHA status (P = 0.02 and P = 0.002, respectively). Whole blood EPA was negatively associated with triacylglycerol (P = 0.003) and positively with total cholesterol, low density and high density lipoprotein (HDL) cholesterol and HDL:triacylglycerol (all P<0.01) whereas DHA was negatively associated with insulin and HOMA-IR (P = 0.003) and tended to be negatively associated with a metabolic syndrome-score (P = 0.05). Adjustment for waist circumference and physical activity did not change the associations. The association between DHA and HOMA-IR was attenuated but remained after adjustment for fiber intake and none of the other associations were confounded by dietary fat, protein, fiber or energy intake. This study showed that EPA status was negatively associated with triacylglycerol and positively with cholesterols whereas DHA was negatively associated with insulin resistance, and both were inversely associated with heart rate in children. The sex-specific associations with blood pressure confirm our previous findings and warrant further investigation.

## Introduction

Intake of n-3 long-chain polyunsaturated fatty acids (LCPUFA) from fish and fish oils have been shown to improve cardiovascular risk markers in adults, most pronouncedly plasma triacylglycerol and blood pressure, and may also reduce coronary heart disease mortality [1], although not all meta analyses agree [2]. The metabolic syndrome (MetS) is defined as a cluster of cardiovascular risk factors including abdominal obesity, dyslipidemia, glucose intolerance, and hypertension [3]. In parallel with the obesity epidemic, increasing numbers of adolescents in the Western world now show features of the MetS [4] and metabolic dysregulations during childhood increase the risk of MetS and type II diabetes in adulthood [5]. However, little is known about the effects of n-3 LCPUFA on cardiometabolic risk markers in school children.

Previous trials conducted by our group showed that fish oil supplementation reduced blood pressure in infants and adolescent boys [6], [7], as has been demonstrated in adults [8]. However, in a cross-sectional pilot study in 73 school children we recently found that docosahexaenoic acid (DHA, 22∶6n-3) in whole-blood was associated with increased blood pressure, but in boys only [9]. Comparable results were seen among the boys of our cross-sectional study of Danish 17-year-olds [10] and in 7-year-old boys of mothers who were supplemented with n-3 LCPUFA during lactation [8]. Overall, this indicates that effects related to n-3 LCPUFA in children may be sex-specific, and that the two major dietary n-3 LCPUFA, eicosapentaenoic acid (EPA, 20∶5n-3) and DHA, may differentially affect the individual components of the MetS cluster in children. In adults, effects on blood pressure seem to be specific to DHA and effects on cholesterols may differ between EPA and DHA [11]. Our previous cross-sectional study among school children showed positive associations with high density lipoprotein (HDL) cholesterol for EPA, not DHA in whole blood [9]. In that study whole blood DHA was negatively associated with physical activity [9]. In the boys of the fish oil-supplemented lactating mothers we also observed lower physical activity and higher energy intake compared to the control group [8], and therefore the increases in blood pressure may have been mediated through reduced physical activity or increased energy intake. On the other hand, consumption of n-3 LCPUFA has also been shown to reduce waist circumference and fat mass [12], which may therefore be potential mediators of any beneficial effects of n-3 LCPUFA on cardiometabolic markers. Finally, n-3 LCPUFA status is positively associated with protein intake [13] and may be linked to total fat intake and to key components in a healthy diet such as fiber [14]. These components have also been associated with cardiometabolic risk markers in children [15], [16], and should therefore be considered as potential confounders when investigating associations between n-3 LCPUFA and early cardiometabolic markers.

The aim of this study was to 1) investigate associations between n-3 LCPUFA status, measured as EPA and DHA in whole blood, and early cardiometabolic risk markers in 8–11-year-old Danish children and 2) explore the potential mediating effects of waist circumference and physical activity and potential dietary confounding.

## Methods

### Study design and participants

The study was based on cross-sectional baseline data from the Optimal well-being, development and health for Danish children through a healthy New Nordic Diet (OPUS) School Meal Study that aimed to investigate the effects of school meals based on the New Nordic Diet on health, well-being, and cognitive performance [17]. The study was conducted according to the guidelines in the Declaration of Helsinki, approved by the Danish National Committee on Biomedical Research Ethics (no. H-1-2010-124), and the baseline study was registered at www.clinicaltrials.gov as NCT01577277. Children from third and fourth grade at nine schools in the Eastern part of Denmark were invited to participate in the study, and the baseline assessments were performed from August to December 2011. Children were excluded only if they had severe food-related allergies, food intolerances, or malabsorption, severe mental handicaps or were participating in other research projects that involved blood sampling or radiation. A total of 1021 children were invited for the school meal study. Hereof, the parents of 834 children (82%) gave written consent for participation, after the study had been explained to the families both orally and in writing [17]. The current paper is based on baseline data from the 713 children from whom information on parental education and pubertal status as well as anthropometric measurements and blood samples were collected.

### Socioeconomic status, diet, and physical activity

The participating families underwent a 2-hour in-depth interview, either at school or in their home, about socioeconomic status and demographics, during which instructions on diet and physical activity recording were given. We defined parental educational level as the level of education of the parent with the highest level in the household, categorized as described by Statistics Denmark [18]. With parental assistance pubertal status was self-evaluated by the child in five categories (Tanner stages) based on breast development in girls and pubic hair in boys [19].

With help from their parents, the children recorded their daily intake of food and beverages every night for 7 consecutive days using a web-based dietary assessment software developed for and validated in 8–11 year-old Danish children [20], [21]. Energy and nutrient intake was calculated using the software system GIES (Version 1.000 d-2010-02-26) developed at the National Food Institute, Technical University of Denmark. Based on reported energy intake and estimated basal metabolic rate (BMR) [22], under reporters (energy intake:BMR≤1.05) (*n* = 55), and over reporters (energy intake:BMR≥2.29) (*n* = 12) [23] were excluded from the dietary analyses.

Physical activity was measured for the same 7 days as the dietary recordings using a tri-axis accelerometer (GT3X or GT3X+, ActiGraph, Pensacola, FL) worn in an elastic belt tightly at the right hip. The children were asked only to remove the accelerometer during water activities, i.e. showering and swimming. Data was reintegrated to 1-min epochs using ActiLife (version 6.0.0, ActiGraph, Pensacola, FL) as previously described [24]. In short, all data obtained between 6 am and midnight was used as wear-time with exclusion of periods of >15 min of consecutive zeros plus wear-time periods <60 min. The child was included only if physical activity was registered for ≥10 hours on ≥3 weekdays and ≥1 weekend day. Total physical activity expressed as counts/min was calculated as the total number of vertical counts divided by wear-time. Time spent on sedentary activity was defined as all minutes showing ≤100 counts/min. Light and moderate-vigorous intensity activity were defined as the numbers of minutes spend with activity in the range of 101–2295 and ≥2296 counts/min, respectively [25]. Median (range) days of valid recording was 5 (3–6) weekdays and 2 (1–2) weekend days with a mean±SD monitor wear time (excluding sleep time) of 901±34 min/day.

### Clinical measurements and blood sampling

Clinical measurements and blood sampling were performed by standard procedures in the morning in an air-conditioned double-decker truck equipped as a mobile laboratory and visiting the schools sequentially. All children, except 22 (8 of whom had only had chewing gum or single bites of food), reported to have fasted overnight except for 1–2 glasses of water. Local anaesthetic patches (EMLA; Astra Zeneca) were provided and venous blood (35–40 mL) was drawn from the antecubital vein. Blood pressure and heart rate were measured by an automated device (UA-787 Plus, A&D Medical) after a 10 min rest using two different cuff sizes (18–22 cm or 22–32 cm). A second device (ProBP 3400 Sure BP; Welch Allyn Inc.) was used for children with arm circumferences <18 cm (*n* = 75). Measurements were performed three times, and the mean of the last two measurements was used. Mean arterial pressure was calculated as (1/3×systolic blood pressure)+(2/3×diastolic blood pressure). Ambient temperature inside the truck was measured by an electronic thermometer. Height was measured three times to the nearest 0.1 cm using a portable stadiometer (CMS Weighing Equipment), with the children holding their heads in the Frankfurt horizontal plane. The mean of all three measurements was used. Body weight was measured to the nearest 0.1 kg on a digital scale (Tanita 800S; Tanita). Children wore light clothing and were asked to empty their bladder prior to measurement. Sex- and age-adjusted z-scores for body mass index (BMI) were calculated using WHO AnthroPlus software [26]. The prevalence of underweight, overweight, and obesity was based on age- and sex-specific cut-offs as described by Cole et al. [27], [28]. Children’s whole-body composition was measured by DXA scan (Lunar Prodigy; GE Medical) using Encore software version 13.5. Most of the children had a standardized breakfast prior to the scanning. Only body fat percentage was used in the present study calculated as fat mass divided by estimated body weight from the scanning (sum of fat mass, lean mass, and bone mass). A continuous MetS score was calculated as the sum of individual continuous Z-scores of the five variables logaritmized: waist circumference, homeostatic model assessment-insulin resistance (HOMA-IR), triacylglycerol, mean arterial pressure, and – HDL cholesterol, as suggested for pediatric populations by Eisenmann [29]. The choice of markers to be included was based on the International Diabetes Federation’s definition of the MetS for children ≥10 years [30], although we included HOMA-IR rather than blood glucose because the latter is highly stable in non-diabetic children, even among obese adolescents with MetS features [31].

### Blood analyses

Whole blood haemoglobin was analysed immediately after sampling on a Hemocue Hb 201 analyzer (Hemocue Denmark). Plasma glucose concentrations were also assessed on fresh blood by a Hemocue Glucose 201 (Hemocue Denmark) calibrated to calculate plasma concentrations from whole blood. Blood collected in serum separation tubes with gel was left to coagulate for 30 min at room temperature and centrifuged at 2500×g for 10 min and the serum stored at −80°C for analysis of insulin. Plasma for measurement of cholesterols and triacylglycerol was obtained from heparinized blood and plasma for measurement of inflammatory markers was obtained from blood with ethylenediaminetetraacetic acid, all by centrifugation at 2500×g for 10 min, and stored at −80°C. Heparinized whole blood was mixed with 0.1% butylated hydroxytoluene (BHT; Sigma-Aldrich) in ethanol (0.1 mL per mL blood) and stored at −80°C for analysis of fatty acid composition. Serum insulin was measured by an automated chemiluminescent immunoassay on an ADVIA Centaur XP (Siemens Healthcare). Insulin concentrations were converted from pmol/L to mIU/L by dividing by 6.945 and HOMA-IR was calculated as plasma glucose (mmol/L) × serum insulin (mIU/L)/22.5 [32]. Plasma total and HDL cholesterol and triacylglycerol were measured on a Vitros 5.1 FS (Ortho-Clinical Diagnostics). Low density lipoprotein (LDL) cholesterol concentrations were calculated by Friedewald’s equation [33]. Plasma interleukin-6 (high sensitive) and adiponectin were measured in duplicate by enzyme-linked immunosorbent assay (R&D Systems). The inter- and intra-assay CV were: 1.4% and 1.2% (total cholesterol); 2.0% and 1.2% (HDL cholesterol); 1.5% and 0.8% (triacylglycerol); 2.5% and 3.1% (insulin); 6.7% and 2.9% (interleukin-6); and 11% and 3.8% (adiponectin). The inter-assay CV was 4.0% for glucose.

Whole blood fatty acid composition was measured by high-throughput gas chromatography within 3 months after blood sampling. Fatty acid methylesters were prepared from whole blood by direct trans-esterification with convectional heat as previously described [34]. Briefly, blood was added to an internal standard (22∶3n-3 ethyl ester; Nu-Check Prep), mixed with 14% BF_3_ in methanol (Pierce Chemicals) and hexane containing 50 µg/mL BHT and convectionally heated for 60 min at 90°C. Fatty acid methylesters were then extracted by addition of water and hexane and the top hexane layer was collected and separated on a Varian 3900 gas chromatograph equipped with a DB-FFAP capillary column (15 m × 0.10 mm i.d. × 0.10 µm film thickness, J&W Scientific; Agilent Technologies) [35]. A total of 97±1% of the chromatogram peaks with retention times between 12∶0 and 22∶6n-3 were identified and the mean total whole blood fatty acids amounted to 225±37 µg/100 µL (range 56–365 µg/100 µL). The amount of individual fatty acids and fatty acid classes are given in weight% of the total whole blood fatty acids. The intra- and inter-assay CV were 1.3% and 4.5% for EPA and 2.4 and 6.4% for DHA, respectively.

### Statistical analysis

Descriptive data are presented as mean ± SD separately for girls and boys and were compared using unpaired t test or Mann-Whitney U test (for non-normally distributed variables). Descriptive associations between whole blood n-3 LCPUFA and fish intake were tested by Pearson’s correlations. Included and excluded children were compared using unpaired t test and chi-square test.

Potential associations between whole blood EPA and DHA status and the cardiometabolic markers were investigated in a mixed linear model including school and class as random effects, parental education, sex and puberty (yes/no) as categorical fixed effects, and age and total concentration of fatty acids in whole blood as covariates. Models of blood pressure and heart rate further included height, ambient temperature in the truck and blood pressure device (small or large) and models of waist circumference, plasma glucose, insulin, and HOMA-IR included height. Models of the MetS score included all of these covariates. If the n-3 LCPUFA-sex interaction term was significant the analysis was performed separately in girls and boys. A sex-pubertal stage interaction term (taking into account that puberty was measured on different scales in boys and girls) were tested in all models.

Secondary analyses were performed to investigate whether associations between n-3 LCPUFA and the cardiometabolic markers were mediated through variations in waist circumference, total physical activity, or energy intake (expressed as energy intake:BMR) or confounded by intakes of protein (expressed as energy%), total fat (expressed energy%) or fiber (expressed as g/10 MJ). In order for a variable to be considered a potential mediator, it had to be associated with EPA or DHA in whole blood and the cardiometabolic outcome in question [36]; this was tested by Pearson’s correlations. The potential mediators were introduced one by one into the basic models described above, based on the difference of coefficients approach procedure for testing mediation in linear regression models [36]. If this weakened the regression coefficient for the association between the n-3 LCPUFA and the cardiometabolic marker >10%, it was interpreted as some degree of mediation. Potential confounders were tested in the mixed models to see if they rendered the association between the n-3 LCPUFA and the cardiometabolic marker non-significant.

Model checking was based on visual inspection of residual and normal probability plots. Insulin, HOMA, triacylglycerol, and adiponectin were logarithmically transformed; interleukin-6 was double-log transformed before analysis and estimates were back-transformed [37]. One girl who had an extreme whole blood EPA value of 4.61 weight%, compared to a median (range) of 0.57 (0.17–1.98) weight% in the rest of the population, was removed from analyses that included EPA. Data were analyzed with SPSS version 20 (IBM Corporation) and R (R Development Core Team) and statistical significance was established at P<0.05.

## Results

### Children’s characteristics

Included children were from households with slightly higher education level than excluded children (P = 0.049, chi-square test) but did not differ with regard to age, sex distribution or degree of overweight (data not shown). Among the included children boys were slightly older, more physically active, and had lower body fat percentage than girls and as expected, more girls than boys had entered puberty (**Table 1**). Among the girls 37% and 9% were in Tanner stage 2 and 3, respectively, whereas these numbers were 21% and 4% among the boys. A total of 632 children (89%) had valid dietary records and 689 children (97%) had valid physical activity recordings. The 81 children with missing or invalid dietary recordings were more likely to be overweight, from a household with low education and had higher HOMA-IR and plasma insulin (data not shown, all P<0.001) than the rest of the study population, but did not differ with regard to age, sex distribution, plasma lipid profile, or whole blood n-3 LCPUFA status.

**Table 1:** Sociodemographic, anthropometric, and lifestyle characteristics of the children.

	Girls (*n* = 343)	Boys (*n* = 370)
Parental education, n (%)		
≤ Lower secondary education	20 (6)	16 (4)
Upper secondary education	13 (4)	7 (2)
Vocational education	113 (33)	116 (31)
Short higher education	33 (10)	37 (10)
Bachelor’s degree or equivalent	96 (28)	110 (30)
≥ Master’s degree	68 (20)	84 (23)
Age, years	9.9±0.7	10.1±0.6**
Pubertal status,% entered puberty^1^	46	25***
Weight, kg^2^	34.8±6.9	35.4±7.2
Height, m	1.42±0.07	1.43±0.07
BMI-for-age z-score^2^	0.07±1.04	0.21±1.11
Weight status, % UW/NW/OW/OB^2^^,^ 3	11.7/74.6/12.0/1.8	8.9/77.8/11.1/2.2
Waist circumference, cm	62.6 (58.9–68.4)	62.4 (59.3–68.2)
Body fat percentage^4^	25.5±7.9	20.6±8.2***
Total physical activity, counts/min^5^	451±117	518±138***
Dietary intake^6^		
Energy, kJ/day	7235±1210	8306±1384***
Protein, energy %	15.1±2.1	15.5±2.0*
Fat, energy %	31.9±3.9	32.3±4.2
Fiber, g/10 MJ	24±5	24±6
Fish and fish products, g/week	80 (0–192)	84 (0–213)
n-3 LCPUFA supplement consumers, n (%)	21 (7)	15 (5)

Values are mean±SD or median (25^th^–75^th^ percentile) unless stated otherwise. BMI, body mass index; LCPUFA, long-chain polyunsaturated fatty acids; NW, normal weight; OB, obese; OW, overweight; UW, underweight.

Different from girls, *P<0.05, **P<0.01, ***P<0.001.

1 Corresponding to self-reported Tanner stage 2 or higher [19].

2 *n* = 342 girls and *n* = 370 boys.

3 Based on age- and sex-specific cut-offs defined by Cole et al. [27], [28].

4 *n* = 342 girls and *n* = 368 boys.

5 *n* = 334 girls and *n* = 356 boys.

6 *n* = 301 girls and *n* = 331 boys.

As shown in Table 1 boys had higher intakes of energy and protein than girls. Among the children 6% reported consumption of dietary supplements containing n-3 LCPUFA during the registration week. Boys had higher relative content of whole blood polyunsaturated fatty acids, due to higher contents of 20∶3n-6, 22∶5n-6, and 22∶5n-3 than girls, whereas no sex differences were seen in the n-6/n-3 ratio (**Table 2**). Whole blood EPA and DHA were associated with each other (r = 0.72, P<0.001). The n-3 LCPUFA were also associated with reported fish intake (r = 0.37 for EPA and r = 0.40 for DHA, both P<0.001).

**Table 2:** Fatty acid composition of children’s whole blood samples.

	Girls (*n* = 343)	Boys (*n* = 370)
	weight%	
Saturated fatty acids	41.72±1.88	41.91±1.98
Monounsaturated fatty acids	21.19±1.60	20.91±1.68
Polyunsaturated fatty acids	33.75±2.84	34.03±2.82*
n-6 polyunsaturated fatty acids	28.64±2.50	28.89±2.48
18∶2n-6	16.03±1.98	15.98±1.95
20∶3n-6	1.52±0.30	1.61±0.30***
20∶4n-6	9.13±1.22	9.27±1.19
22∶4n-6	1.24±0.23	1.26±0.23
22∶5n-6	0.28±0.08	0.30±0.08*
n-3 polyunsaturated fatty acids	5.11±1.09	5.14±1.07
18∶3n-3	0.29 (0.25–0.35)	0.28 (0.24–0.34)
20∶5n-3 eicosapentaenoic acid	0.56 (0.42–0.71)^1^	0.57 (0.45–0.77)
22∶5n-3	1.17±0.18	1.23±0.18***
22∶6n-3 docosahexaenoid acid	3.00±0.76	2.96±0.74
n-6/n-3 polyunsaturated fatty acids	5.9±1.3	5.9±1.3

Values are mean±SD or median (25^th^–75^th^ percentile). Different from girls, *P<0.05, ***P<0.001.

1 *n* = 342 girls for EPA, due to one extreme outlier.

Boys had lower diastolic blood pressure, heart rate, serum insulin, HOMA-IR, plasma triacylglycerol, adiponectin, and interleukin-6 and higher plasma glucose, HDL cholesterol, and HDL: triacylglycerol than girls (**Table 3**). Accordingly, the MetS score was more than 1 z-score lower in the boys compared to the girls.

**Table 3:** Cardiometabolic risk markers in the children.

	Girls *(n* = 343)	Boys (*n* = 370)
Systolic blood pressure, mmHg	107±9	108±8
Diastolic blood pressure, mmHg	69±7	67±6**
Mean arterial pressure, mmHg	82±7	81±6
Heart rate, beats/min	81±11	76±11***
Glucose, mmol/L	5.1±0.5	5.3±0.4***
Insulin, pmol/L	45 (34–63)	39 (30–54)***
HOMA-IR	1.52 (1.09–2.10)	1.34 (0.98–1.89)**
Total cholesterol, mmol/L	4.08±0.63	4.07±0.62
LDL cholesterol, mmol/L	2.35±0.55	2.30±0.55
HDL cholesterol, mmol/L	1.39±0.28	1.49±0.3***
Triacylglycerol, mmol/L	0.66 (0.54–0.84)	0.57 (0.48–0.70)***
HDL : triacylglycerol	2.14±0.90	2.61±0.98***
Metabolic syndrome score	0.54±3.30	−0.53±2.82***
Adiponectin, µg/L^1^	11,289 (8,371–14,946)	10,531 (7,743–14,062)*
Interleukin-6, ng/L^2^	0.97 (0.69–1.62)	0.79 (0.57–1.09)

Values are mean±SD or median (25^th^–75^th^ percentile). HDL, high density lipoprotein; HOMA-IR, homeostatic model assessment-insulin resistance; LDL, low density lipoprotein.

^*^Different from girls, *P<0.05, **P>0.01, ***P<0.001.

1 *n* = 338 girls and *n* = 367 boys.

2 *n* = 342 girls and *n* = 368 boys.

### Associations between whole blood n-3 LCPUFA and cardiometabolic risk markers

**Figure 1:**
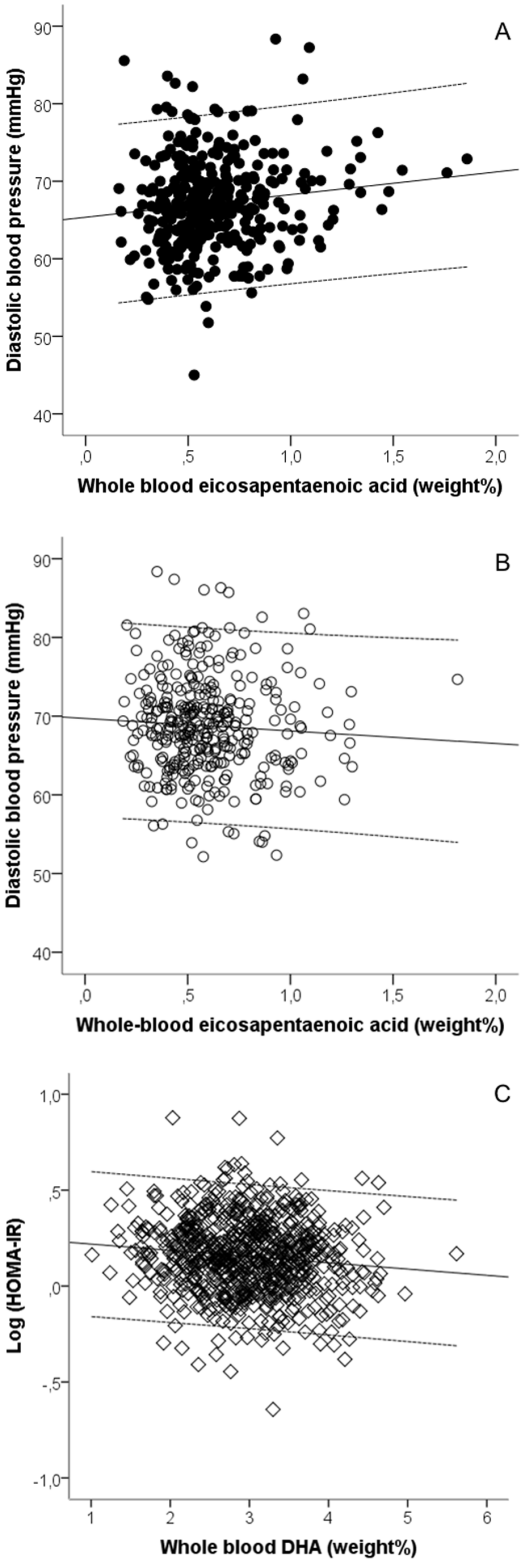
Whole blood eicosapentaenoic acid is sex-specifically associated with diastolic blood pressure whereas docosahexaenoic acid is negatively associated with HOMA-IR in all children. Regression lines and 95% CI are shown for the associations between eicosapentaenoic acid and diastolic blood pressure in boys, *β* = 2.9, *P* = 0.02, *n* = 366 (A) and girls, *β* = −1.6, *P* = 0.28, *n* = 340 (B) and for the association between docosahexaenoic acid and HOMA-IR in the total study population, *β* = −0.03, *P* = 0.002, *n* = 713 (C). Plots were adjusted for school, class, parental education, age, height, puberty, and total fatty acids in whole blood. Blood pressure plots were additionally adjusted for ambient temperature and blood pressure device.

Diastolic blood pressure was positively associated with EPA status in boys, whereas no association was seen in girls (**Table 4** and **Figure 1A and 1B**). Heart rate was negatively associated with whole blood EPA and DHA in the sexes combined (Table 4). EPA was positively associated with all plasma cholesterols, but negatively with plasma triacylglycerol and therefore positively associated with the HDL: triacylglycerol ratio whereas DHA was negatively associated with plasma insulin and HOMA-IR (Table 4 and **Figure 1C**). DHA status was also negatively associated with plasma triacylglycerol, but in girls only. The MetS score tended to be negatively associated with whole blood DHA (P = 0.052) (Table 4). A borderline significant sex-EPA interaction was seen in the MetS score (P = 0.056) and when the analysis was conducted sex-specifically it showed a negative association between EPA and the MetS score in girls [β = −1.42 (95% CI −2.65; −0.19)] (P = 0.024, n = 340) and no association in boys [β = 0.01 (95% CI −0.97; 0.99)] (P = 0.99, n = 366).

**Table 4:** Associations between whole blood n-3 long-chain polyunsaturated fatty acids and cardiometabolic risk markers in the children.

	Eicosapentaenoic acid, weight%			Docosahexaenoic acid, weight%		
	*n*	*β* (95% CI)	*P* value	*n*	*β* (95% CI)	*P* value
Waist circumference	712	1.7 (−0.14; 3.5)	0.07	713	0.09 (−0.54; 0.73)	0.78
BMI-for-age z-score	711	0.16 (−0.14; 0.45)	0.30	712	−0.02 (−0.11; 0.09)	0.79
Body fat%	709	1.6 (−0.6; 3.8)	0.17	710	0.04 (−0.74; 0.82)	0.92
Systolic blood pressure, mmHg	706	−1.2 (−3.4; 1.1)	0.31	707	−0.6 (−1.4; 0.1)	0.11
Diastolic blood pressure, mmHg	340 (F)366 (M)	−1.6 (−4.4; 1.2) (F)*2.7 (0.4; 5.1) (M)	0.27 (F)0.02 (M)	707	0.0 (−0.6; 0.7)	0.95
Mean arterial pressure, mmHg	706	0.16 (−1.6; 1.9)	0.86	707	−0.2 (−0.8; 0.4)	0.51
Heart rate, beats/min	706	−3.8 (−6.9; −0.6)	0.02	707	−1.8 (−2.9; −0.7)	0.002
Glucose, mmol/L	712	−0.03 (−0.15; 0.10)	0.69	713	−0.04 (−0.08; 0.01	0.10
Insulin, mmol/L	712	−4.45 (−9.88; 0.97)	0.11	713	−2.85 (−4.75; −0.95)	0.003
HOMA-IR	712	−0.16 (−0.35; 0.04)	0.12	713	−0.10 (−0.17; −0.03	0.003
Total cholesterol, mmol/L	712	0.26 (0.09; 0.43)	0.003	713	0.02 (−0.04; 0.08)	0.46
LDL cholesterol, mmol/L	712	0.23 (0.07; 0.38)	0.004	713	0.04 (−0.01; 0.10)	0.11
HDL cholesterol, mmol/L	712	0.11 (0.03; 0.20)	0.009	713	0.01 (−0.03; 0.03)	0.76
Triacylglycerol, mmol/L	712	−0.09 (−0.15; −0.03)	0.003	343 (F)370 (M)	−0.04 (−0.08; −0.01) (F)*−0.01 (−0.03; 0.02) (M)	0.02 (F) 0.59 (M)
HDL : triacylglycerol	712	0.39 (0.13; 0.66)	0.003	713	0.04 (−0.05; 0.13)	0.39
Metabolic syndrome score	706	−0.68 (−1.45; 0.10)	0.09	707	−0.27 (−0.55; 0.00)	0.05
Adiponectin, µg/L	704	504 (−974; 1983)	0.49	705	235 (−285; 755)	0.37
Interleukin-6, ng/L	709	0.12 (−0.04; 0.29)	0.15	710	0.011 (−0.02; 0.04)	0.55

Values are slope coefficients (95% CI) for the association between the fatty acids and the cardiometabolic markers linear mixed models adjusted for school, class, parental education, sex, puberty, age, and total concentration of fatty acids in whole blood. Blood pressure and heart rate were further adjusted for height, ambient temperature and blood pressure device. Waist circumference, plasma glucose, insulin, and HOMA-IR were further adjusted for height, and the metabolic syndrome score were adjusted for all of the above. If there was significant n-3 LCPUFA-sex interaction the analysis was performed in the sexes separately. BMI, body mass index; F, female; HDL, high density lipoprotein; HOMA-IR, homeostatic model assessment-insulin resistance; LCPUFA; long-chain polyunsaturated fatty acids; LDL, low density lipoprotein; M, male.

*Interaction between sex and the n-3 LCPUFA, P-interaction<0.05.

### Potential mediation or confounding of the associations between n-3 LCPUFA and cardiometabolic risk markers

Neither waist circumference (P>0.57), total physical activity (P>0.72) nor energy intake:BMR (P>0.11) were associated with whole blood EPA or DHA in pairwise correlations, and were therefore judged not to be mediators of the demonstrated associations between the n-3 LCPUFA and the cardiometabolic markers. Moreover, waist circumference, BMI z-score, and body fat% were not associated with either EPA or DHA in mixed models (Table 4) and neither of the n-3 LCPUFA were correlated with time spent on sedentary, light, or moderate-vigorous activity (data not shown). Total physical activity was beneficially correlated with all investigated cardiometabolic outcomes except plasma total and LDL cholesterol and adiponectin (data not shown). Also, waist circumference was negatively associated with all cardiometabolic outcomes except for heart rate and total cholesterol (data not shown). Adjustment for protein intake (**Table S1** and **Table S2**) or energy% from total fat (data not shown) as potential confounders did not weaken the associations between EPA or DHA status and the cardiometabolic risk markers. Adjustment for fiber intake as a potential confounder weakened but did not remove the beneficial association between DHA and HOMA-IR (Table S2). In the mixed models where the potential confounders were significant, fiber intake was negatively associated with total and HDL cholesterol (Table S1) and HOMA-IR (Table S2), whereas protein intake was negatively associated with triacylglycerol but positively associated with HOMA-IR (Table S2).

## Discussion

This study confirmed our previous pilot study findings [38] of negative associations between whole blood EPA and triacylglycerol, positive associations between EPA and cholesterols as well as sex-specific associations between the n-3 LCPUFA status and blood pressure in 8–11-year-old Danish children. However, in contrast with our previous findings [38] the present study showed that EPA was the strongest predictor of blood pressure and that both n-3 LCPUFA were negatively associated with heart rate and DHA with insulin resistance. None of the demonstrated associations were statistically mediated by waist circumference or physical activity, and although fiber intake weakened the association between whole blood DHA and insulin regulation, the associations did not seem to be confounded by key dietary components.

In randomized trials n-3 LCPUFA, particularly EPA, consistently lower triacylglycerol in adults [39] but to our knowledge such effects have not been clearly demonstrated in children. The negative association between EPA, not DHA status, and triacylglycerol seem to support the findings in adults although our data are cross-sectional. In contrast to a recent review by Mozaffarian and Wu examining the evidence of differential effects of EPA and DHA on cardiovascular end-points and risk markers in adults [11] both EPA and DHA were negatively associated with heart rate in the present study.

The effect of n-3 LCPUFA on insulin regulation is controversial. Most studies in adults show no effect [40], but this has not been thoroughly investigated in children. A Norwegian case-control study showed lower risk of type 1 diabetes in the offspring of mothers taking cod liver oil during pregnancy, also after adjustment for education and other potential confounders [41]. Also, consistent with the findings of the present study an Australian study showed that insulin and HOMA-IR were negatively associated with EPA+DHA in erythrocytes of obese 5–12-year-old children [42]. In that study the degree of obesity was negatively associated with n-3 LCPUFA status, which we did not find.

In hypertensive adults n-3 LCPUFA supplementation in the form of fish oil consistently lowers blood pressure [43], and we have previously demonstrated this effect in infants and slightly overweight adolescent boys [6], [7]. However, the effects of habitual n-3 LCPUFA intake and status may differ from those of supplementary high-dose fish oil. In the present study n-3 LCPUFA status was positively associated with blood pressure in boys only. This is consistent with our previous findings in a cross-sectional pilot study of Danish 8–11-year-olds [38] and in 7-year-old offspring of mothers who received fish oil versus olive oil supplements during lactation [8]. This could be hypothesized to be related to behavioral effects potentially acting through the central nervous system in a sex-specific manner. In support of this hypothesis, n-3 LCPUFA has been shown to lower locomotor activity in mice [44] and there is some evidence that n-3 LCPUFA can ameliorate symptoms in children with attention-hyperactivity deficit disorders [45] and reduce aggressive behavior in stressed students [46]. However, in contrast with our pilot study observations [38], the present study showed no associations between n-3 LCPUFA status and physical activity. The measurement of blood pressure at only one occasion and shortly before blood sampling is a limitation of the present study and makes it questionable whether the measurements reflect resting or rather stressed conditions. Therefore, whether the observed sex-specific associations reflect a cardiovascular or rather a psychosomatic sex difference in the associations with EPA remains unclear.

The present study is the first to assess associations between n-3 LCPUFA status and a wide range of cardiometabolic markers in healthy children. It is strengthened by the large sample size, elaborate measurements including 7-day recordings of diet and physical activity by accelerometry, as well as clinical measurements and blood samples collected under highly standardized conditions. As part of the explorative yet hypothesis-based approach we conducted multiple statistical tests, which increase the risk of chance findings. Bonferroni corrections are known to be very conservative and not suited for highly correlated data, such as the cardiometabolic markers, as it assumes independent outcomes [47], and were therefore not applied. Therefore the results should be interpreted with caution. However, the tests were based on specific biological hypotheses, we thoroughly adjusted for confounding factors and all these analyses showed highly consistent results. For the dietary data energy under- and over-reporters were excluded based on common cutoffs for all children, assuming a relatively low mean physical activity level (PAL) of 1.55 [23]. This conservative approach was chosen based on its high specificity, on the cost of the sensitivity, which is a limitation of the present study. More individualized cut-offs could have been estimated using each child’s accelerometry data to estimate activity levels. However, the conversion of accelerometer counts to PAL in individual children can be discussed [48] and activities such as swimming and particularly bicycling which is common in Danish children are not captured by the accelerometers. Also, not all children had complete accelerometry data, and we therefore chose a more simple approach.

Our study population was to a large degree representative of Danish children; all educational groups were represented, the baseline diet of the children was in line with the representative Danish National Dietary Surveys [49], and the prevalence of overweight and obesity corresponds to the level found in a recent Danish cohort [50]. However, when trying to determine the effects of n-3 LCPUFA on cardiometabolic risk markers in children, the results should be interpreted with caution as the data presented are cross-sectional and cannot determine causality. However, at least reverse causality seems unlikely i.e. that the cardiometabolic risk markers affect whole-blood n-3 LCPUFA status.

We used whole blood EPA and DHA as markers of n-3 LCPUFA status. In normo-lipidemic individuals erythrocytes, plasma, and buffy coat (which is mainly leucocytes) have been shown to account for approximately 30–35, 50–60, and 10–15% of the total fatty acids in whole blood (K. D. Stark and A. H. Metherel, Department of Kinesiology, University of Waterloo, Ontario, Canada, unpublished results). The response of whole blood fatty acids to n-3 LCPUFA supplementation has been shown to be intermediate between that of plasma (which reflect intakes over the last days or weeks) and erythrocyte fatty acids (reflecting intakes over the last months) [51]. Although EPA and DHA measured in erythrocytes would be a better marker of longer-term intakes, whole blood was a well-suited analysis material and elaborate erythrocyte washing was not required. DHA in blood tends to be mainly located in inner membranes (erythrocytes), whereas EPA tends to be mainly found in the more dynamic outer membranes (lipoproteins and erythrocytes) [51]. In line with this, plasma EPA compared to plasma DHA has been shown to respond faster to the immediate intake of n-3 polyunsaturated fatty acids [52] and this is possibly also true in erythrocytes [53]. Whole blood EPA and DHA were found to correlate strongly with each other. Therefore, we do not know whether the differential associations between whole blood EPA and DHA and some cardiometabolic risk markers are a consequence of different molecular mechanisms of action, a specific sensitivity of EPA and DHA to different ranges of intake, or whether EPA and DHA are markers of different pools of n-3 LCPUFA in the whole blood.

The implications of blood pressure, lipid profile, and insulin in school-aged children for later risk of cardiovascular disease is uncertain. However, atherosclerosis is a gradual and life-long process, blood pressure and lipid profile show tracking from childhood and adolescence to adulthood [54], [55], and metabolic dysregulations during childhood have been shown to increase the risk of MetS and type II diabetes in adulthood [5]. Therefore, it is likely that low values of these markers in childhood will be beneficial over the life course. The implications of positive associations between blood pressure and EPA status in boys are unknown and may not be beneficial, but may depend on the mechanisms behind this association i.e. whether it is a behavioral or a cardiovascular phenomenon.

In conclusion, this study showed that EPA in whole-blood was negatively associated with heart rate and plasma triacylglycerol and positively associated with cholesterols and with blood pressure in boys only, whereas DHA was negatively associated with heart rate and insulin resistance in 8–11-year-old children. The associations were not mediated through waist circumference or physical activity and persisted after adjustment for potential dietary confounders. The sex-specific associations with blood pressure confirm our previous findings and warrant further investigation in long-term randomized controlled trials.

## Supporting Information

Table S1 **Potential dietary confounding of the associations between whole blood eicosapentaenoic acid (weight%) and cardiometabolic risk markers in the children.**
(DOCX)

Table S2 **Potential dietary confounding of the associations between whole blood docosahexaenoic acid (weight%) and cardiometabolic risk markers in the children.**
(DOCX)
