# Supplementary material for: Eicosapentaenoic Acid and Docosahexaenoic Acid in Whole Blood Are Differentially and Sex-Specifically Associated with Cardiometabolic Risk Markers in 8–11-Year-Old Danish Children
Source: PLoS One. 2014 Oct 15;9(10):e109368. doi: 10.1371/journal.pone.0109368 (PMC4198100; doi:10.1371/journal.pone.0109368)
Supplement: Table S1 — Potential dietary confounding of the associations between whole blood eicosapentaenoic acid (weight%) and cardiometabolic risk markers in the children. (DOCX) [file pone.0109368.s001.docx]

**ONLINE SUPPORTING INFORMATION**

**Table S1. Potential dietary confounding of the associations between whole blood eicosapentaenoic acid (weight%) and cardiometabolic risk markers in the children**

| Potential confounder |  | None | |  | Protein intake (energy %) | |  | Fiber intake (g/10 MJ) | |
| --- | --- | --- | --- | --- | --- | --- | --- | --- | --- |
|  | *n* | *β* (95% CI) | *P* value |  | *β* (95% CI) | *P* value |  | *β* (95% CI) | *P* value |
| Diastolic blood pressure, mmHg | 298 (F)  329 (M) | -2.0 (-5.0; 0.91) (F)  3.2 (0.8; 5.7) (M) | 0.17 (F)  0.01 (M) |  | -2.1 (-5.0; 0.9) (F)  3.2 (0.7; 5.6) (M) | 0.17 (F)  0.01 (M) |  | -1.8 (-4.8; 1.1) (F)  3.3 (0.8; 5.7) (M) | 0.23 (F)  0.01 (M) |
| Total cholesterol, mmol/L | 631 | 0.25 (0.07; 0.44) | 0.006 |  | 0.25 (0.06; 0.43) | 0.008 |  | 0.27 (0.09; 0.45) | 0.004^1^ |
| LDL cholesterol, mmol/L | 631 | 0.22 (0.06; 0.39) | 0.007 |  | 0.21 (0.05; 0.37) | 0.011 |  | 0.23 (0.07; 0.39) | 0.005 |
| HDL cholesterol, mmol/L | 631 | 0.12 (0.03; 0.21) | 0.01 |  | 0.12 (0.03; 0.21) | 0.009 |  | 0.13 (0.04; 0.22) | 0.006^1^ |
| Triacylglycerol, mmol/L | 631 | -0.12 (-0.18; -0.06) | <0.001 |  | -0.12 (-0.18; -0.05) | <0.001 |  | -0.12 (-0.19; -0.06) | <0.001 |
| HDL : triacylglycerol | 631 | 0.50 (0.22; 0.78) | <0.001 |  | 0.49 (0.21; 0.77) | 0.001 |  | 0.52 (0.24; 0.80) | <0.001 |

Values are slope coefficients (95% CI) for the association between the fatty acids and the cardiometabolic risk markers in adjusted linear mixed models. If there was significant EPA-sex interaction the analysis was performed in the sexes separately. Only children with valid dietary records were included. EPA, eicosapentaenoic acid; F, female; HDL, high density lipoprotein; LDL, low density lipoprotein; M, male.

^1^Potential confounder significant in the model, *P*<0.05.
