# Supplementary material for: Eicosapentaenoic Acid and Docosahexaenoic Acid in Whole Blood Are Differentially and Sex-Specifically Associated with Cardiometabolic Risk Markers in 8–11-Year-Old Danish Children
Source: PLoS One. 2014 Oct 15;9(10):e109368. doi: 10.1371/journal.pone.0109368 (PMC4198100; doi:10.1371/journal.pone.0109368)
Supplement: Table S2 — Potential dietary confounding of the associations between whole blood docosahexaenoic acid (weight%) and cardiometabolic risk markers in the children. (DOCX) [file pone.0109368.s002.docx]

**ONLINE SUPPORTING INFORMATION**

**Table S2. Potential dietary confounding of the associations between whole blood docosahexaenoic acid (weight%) and cardiometabolic risk**  **markers in the children**

| Potential confounder | None | | |  | Protein intake (energy %) | |  | Fiber intake (g/10 MJ) | |
| --- | --- | --- | --- | --- | --- | --- | --- | --- | --- |
|  | *n* | β (95% CI) | *P* value |  | β (95% CI) | *P* value |  | β (95% CI) | *P* value |
| Heart rate, beats/min | 628 | -1.7 (-2.8; -0.6) | 0.004 |  | -1.6 (-2.7; -0.4) | 0.008 |  | -1.4 (-2.5; -0.2) | 0.022 |
| Insulin, mmol/L | 632 | -2.37 (-4.31; -0.42) | 0.017 |  | -2.78 (-4.75; -0.82) | 0.006^1^ |  | -1.89 (-3.86; 0.07) | 0.06^2^ |
| HOMA-IR | 632 | -0.09 (-0.16; -0.02) | 0.013 |  | -0.10 (-0.18; -0.03) | 0.004^1^ |  | -0.07 (-0.14; -0.00) | 0.047^1^ |
| Triacylglycerol, mmol/L | 301 (F)  331 (M) | -0.05 (-0.08; -0.01) (F)  -0.01 (-0.04; 0.02) (M) | 0.01 (F)  0.39 (M) |  | -0.05 (-0.08; -0.01) (F)  -0.01 (-0.03; 0.02) (M) | 0.008 (F)  0.75 (M)^2^ |  | -0.05 (-0.08; -0.02) (F)  -0.01 (-0.04; 0.02) (M) | 0.006 (F)  0.36 (M) |

Values are slope coefficients (95% CI) for the association between the fatty acids and the cardiometabolic markers in adjusted linear mixed models.

If there was significant DHA-sex interaction the analysis was performed in the sexes separately. Only children with valid dietary records were included. DHA; docosahexaenoic acid; F, female; HOMA-IR, homeostatic model assessment-insulin resistance; M, male.

^1^Potential confounder significant in the model, P<0.05.

^2^Potential confounder significant in the model, P<0.01.
